# Nuclear accumulation of CDH1 mRNA in hepatocellular carcinoma cells

**DOI:** 10.1038/oncsis.2015.11

**Published:** 2015-06-01

**Authors:** S Ghafoory, A Mehrabi, M Hafezi, X Cheng, K Breitkopf-Heinlein, M Hick, M Huichalaf, V Herbel, A Saffari, S Wölfl

**Affiliations:** 1 Institute of Pharmacy and Molecular Biotechnology, Ruprecht-Karls-University Heidelberg, Heidelberg, Germany; 2 Department of General, Visceral and Transplantation surgery, Ruprecht-Karls-University Heidelberg, Heidelberg, Germany; 3 Molecular Hepatology—Alcohol Associated Diseases, II. Medical Clinic, Faculty of Medicine at Mannheim, Ruprecht-Karls-University Heidelberg, Mannheim, Germany

## Abstract

Expression of E-cadherin has a central role in maintaining epithelial morphology. In solid tumors, reduction of E-cadherin results in disruption of intercellular contacts. Consequently, cells lose adhesive properties and gain more invasive mesenchymal properties. Nevertheless, the mechanism of E-cadherin regulation is not completely elucidated. Here we analyzed the distribution of E-cadherin expression at the cell level in human hepatocellular carcinoma, in which human liver paraffin blocks from 25 hepatocellular carcinoma patients were prepared from cancerous (CA) and noncancerous areas (NCA). *In situ* hybridization (ISH) was performed to detect E-cadherin and hypoxia-induced factor-1α (HIF1α) mRNAs and immunohistochemistry to stain E-cadherin protein. In parallel, RNA was extracted from CA and NCA, and E-cadherin and HIF1α were quantified by quantitative reverse transcription PCR. ISH revealed abundant E-cadherin mRNA in nuclei of hepatocellular carcinoma cells (HCCs), whereas immunohistochemistry showed depletion of E-cadherin protein from these areas. In sections of NCA, E-cadherin mRNA was also found in the cytosol, and E-cadherin protein was detected on the membrane of cells. Experiments in cell lines confirmed E-cadherin mRNA in nuclei of cells negative for E-cadherin protein. HIF1α expression is elevated in CAs, which is associated with a clear cytosolic staining for this mRNA. Our results demonstrate that E-caderhin mRNA is selectively retained in nuclei of HCCs, whereas other mRNAs are still exported, suggesting that translocation of E-cadherin mRNA from nuclei to cytoplasm has a role in regulating E-cadherin protein levels during epithelial mesenchymal transition.

## Introduction

E-cadherin (CDH1) is a member of the cadherin superfamily, and it is required for epithelial cell–cell adhesion.^1^ The extracellular domain of E-cadherin consists of five repeated domain units and specifically binds E-cadherin on neighboring cells. Cadherins also provide a link to the cytoskeleton through a transmembrane domain and a cytoplasmic domain binding to beta-actin.^1^ In many solid tumors of epithelial origin, expression of E-cadherin is suppressed, leading to reduced E-cadherin protein levels in cell membranes and consequently weaker cell–cell interactions.^2, 3, 4, 5, 6^ The loss of E-cadherin is a hallmark of cells undergoing an epithelial mesenchymal transition (EMT). Cells lacking E-cadherin in the cell membrane are susceptible to detach and be released from the tumor tissue. EMT is considered to be the first step for dissemination of tumor cells into other areas and consequently for the formation of metastases.^7, 8^ It is also known that hypoxia can stimulate EMT. During tumor growth, rapid cell division without concomitant blood vessel formation leads to the formation of different areas of nutrient and oxygen supply, which results in different growth conditions for cancer cells in the tumor.^4, 9, 10^ Although in the marginal zone access to oxygen and nutrients is available, in the center of the tumor hypoxic condition prevails. As a result, hypoxia-induced factor-1α (HIF1α) is upregulated, which can induce angiogenesis factors such as VEGF^9, 11^ but also further promote EMT, which besides downregulation of E-cadherin also leads to expression of EMT-specific genes, for example, Vimentin, Twist, Slug and Zeb1.^9, 10^ The resulting reduced cell adhesion to neighboring cells allows cancer cells to migrate and escape from hypoxia conditions in the center of the tumor, and in consequence disseminated cancer cells can lead to the formation of metastases.^4, 9, 12^

Among different cancers, hepatocellular carcinoma is one of the most common worldwide malignancies with a rising incidence in western countries, and it is the second leading cause of cancer death.^13^

It is unclear how downregulation of E-cadherin occurs at the level of individual cells within the tumor tissue; therefore, we decided to analyze the expression of E-cadherin (CDH1) in hepatocellular carcinomas by *in situ* hybridization (ISH) and fluorescent immunohistochemistry (FIHC) of liver sections from patients, using matched tissue sections for cancerous (CA) and noncancerous (NCA) tissue areas.

Although in the NCAs the E-cadherin protein was clearly detected in the cell membrane of hepatocytes, no signal was obtained in CAs by FIHC. To our surprise, a different picture emerged in ISH experiments, in which high signals for CDH1 mRNA were visible in the CAs. Closer examination revealed, however, that the ISH signal for CDH1 in the CAs was confined to nuclei with no visible cytoplasmic mRNA staining. In cells located in NCAs, mRNA staining was obtained in both nucleus and cytoplasm, but staining of the nuclei was much weaker in comparison with the signal in CAs. These results suggest a retention of E-cadherin mRNA in the nucleus, which could contribute to the cancer-associated downregulation of E-cadherin protein in the cell membrane. We compared these observations with the expression of HIF1α on mRNA and protein level in hepatocellular carcinoma cell (HCC) tissues, which is known to remain highly expressed in hypoxic conditions found in cancerous tissue. To further evaluate the observed expression pattern, we also analyzed overall E-cadherin (CDH1) mRNA levels in matching tissue pairs by quantitative reverse transcription PCR (RT–qPCR) and analyzed intracellular mRNA distribution in the hepatoma cell line HLE and in the epithelial breast cancer cell line MCF7. The observed retention of CDH1 mRNA in the cell nucleus of HCCs, while other mRNAs like albumin and HIF1α are still efficiently transported and expressed, led us to conclude that there is a more general mechanism that allows selective retention of CDH1 mRNAs in the nucleus, while other mRNAs are still transported and translated, which could contribute to the downregulation of E-cadherin protein levels in HCCs.

## Results

### Expression of E-cadherin (CDH1) in liver sections of HCC patients

**Figure 1:**
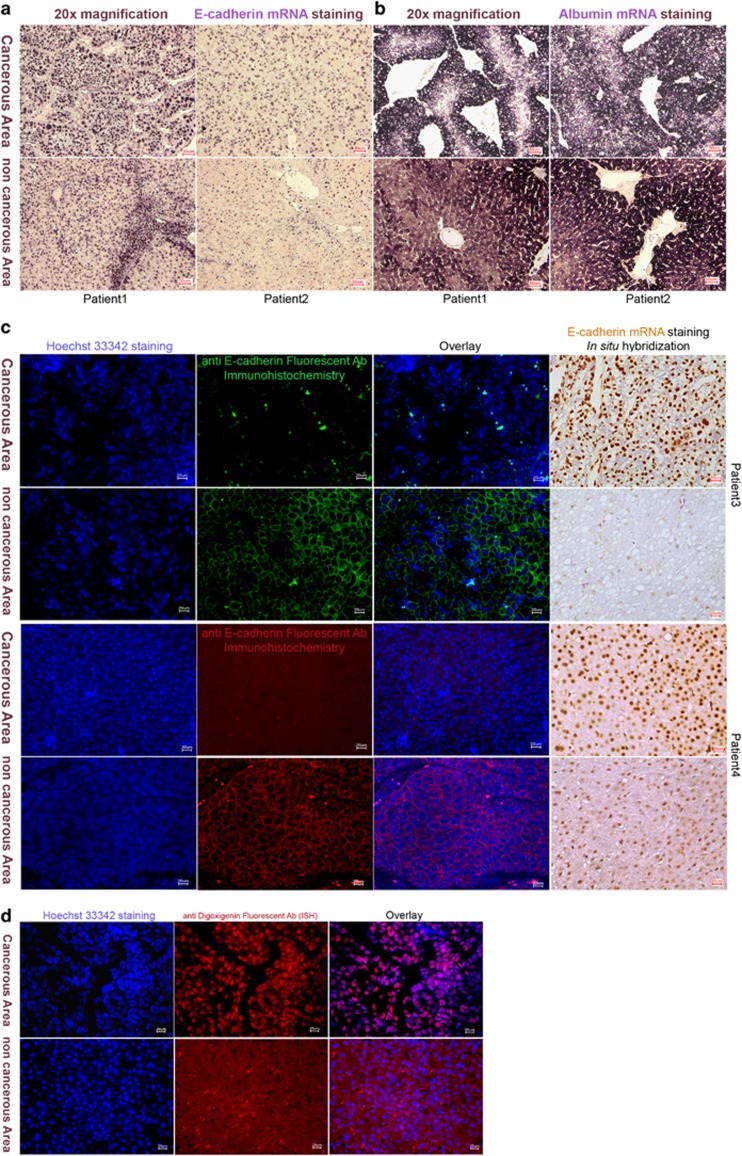
E-cadherin expression in hepatocellular carcinoma tissues. (**a**) CDH1 mRNA staining (violet) in CAs and NCAs from two HCC patients. In CAs, CDH1 mRNA is only detected in the nucleus; in NCAs, CDH1 mRNA staining is visible in both nucleus and cytosol. (**b**) Hybridization (violet) for Albumin mRNA, a liver-specific gene highly expressed in hepatocytes. Albumin mRNA is detected in CAs and NCAs of the same HCC patients (as in **a**). The staining (violet) for albumin mRNA is stronger in the cytoplasm of both CAs and NCAs. (**c**) Detection of E-cadherin at the protein level using two different primary and secondary antibodies (green or red) and overlay with mRNA staining (brown) in two HCC patients. Loss of E-cadherin protein staining in membranes of cells in CAs correlates with high nuclear CDH1 mRNA levels, whereas in NCAs a clear membranous staining pattern of E-cadherin correlates with a clear cytosolic staining of CDH1 mRNA and a weaker nuclear signal. (**d**) Fluorescence ISH using an anti-DIG-Cy3 antibody for the detection of hybridized antisense E-cadherin probes in CAs and NCAs from one patient. Nuclei were visualized by Hoechst staining (blue). The overlay between blue (nuclei) and red (CDH1 mRNA) fluorescence corroborates CDH1 mRNA retention in nuclei in CAs, whereas in NCAs CDH1 mRNA is mostly located in the cytoplasm.

To identify potential local differences of E-cadherin (CDH1) expression in liver sections of HCC patients, we prepared ISH probes for the detection of CDH1 mRNA, following our rapid protocol for the preparation of antisense RNA probes.^14^ These probes were subsequently used to visualize CDH1 mRNA distribution in complementary liver sections from HCC patients representing CAs and NCAs. To our surprise, strong signals were obtained in the cancerous HCC tissue sections (Figure 1a and Supplementary Figure S1). Closer inspection of the images clearly showed that the staining for CDH1 mRNA is selectively nuclear in the CAs of the tissue sections. As ISH for CDH1 mRNA with sections of NCAs also show some nuclear staining, tissue sections from each patient of both CAs and NCAs were mounted on the same slide to ensure equal experimental handling during hybridization and processing. In addition, pictures were taken with a digital microscope using identical settings. Under this controlled condition, it can be clearly seen that in NCA sections CDH1 mRNA was also clearly visible in the cytosol, whereas in all tumorous tissue areas staining for CDH1 mRNA was completely shifted to the nucleus (Figure 1a and Supplementary Figure S1). Similar staining patterns were obtained with all matched CA and NCA tissue sections of HCC patients analyzed.

We then asked whether this nuclear staining could be a specific property of the strongly proliferating tumor cells and specific for CDH1. Thus, we next analyzed the distribution of albumin mRNA, which is abundantly expressed in hepatocytes,^15^ in consecutive sections, processed in the same way as above. Using probes for albumin mRNA (Figure 1b and Supplementary Figure S1), no nuclear staining pattern was obtained. The albumin-specific probes clearly stained cytosolic areas and did not show an increase in nuclear staining in both CA and NCA tissue sections (Figure 1b and Supplementary Figure S1). Thus, the nuclear pattern observed for CDH1 mRNA in the cancerous tissue was very specific for this gene.

To further rule out that an experimental artifact led to the staining pattern obtained for CDH1, we also performed ISH with a fluorescence staining protocol using a Cy-3-labeled anti-digoxigenin antibody, and repeated the analysis with randomly selected sections of two patients (Figure 1d). The fluorescence images obtained showed a similar cellular distribution of the E-cadherin mRNA as seen with our standard protocol, that is, strong nuclear staining but no staining in the cytoplasm of cells in CAs, and a clear cytoplasmic signal for E-cadherin mRNA in NCA tissue sections (Figure 1d). These results confirmed that the observed change in the subcellular localization of E-cadherin mRNA is very specific for this gene and not a staining artifact occurring in CA cells. We therefore concluded that, in cells of the CAs, E-cadherin mRNA is specifically retained in the nuclei, whereas in NCAs E-cadherin mRNA is transported to the cytoplasm (Figures 1a, d and c last column).

### Loss of E-cadherin protein in tumor tissue is correlated with retention of CDH1 mRNA

We next asked whether the retention of the mRNA in the nucleus could correlate with E-cadherin protein levels. Therefore, both E-cadherin mRNA and protein levels (for each patient) were analyzed in subsequent sections (Figure 1c). To ensure specificity in FIHC, we used two different anti-E-cadherin monoclonal antibodies. In the analysis of subsequent sections by FIHC and ISH, it can be clearly seen that the strong nuclear signal for CDH1 mRNA correlates with a strong reduction or even a complete loss of E-cadherin protein staining in cell membranes. In corresponding experiments with NCA sections, a strong staining for E-cadherin protein in cell membranes correlates with a weak nuclear and clear cytoplasmic staining for CDH1 mRNA. Thus, the observed nuclear retention of CDH1 mRNA correlates with low E-cadherin protein levels and implies that transport of the CDH1 mRNA from the nucleus to the cytoplasm may be a regulated process participating in the control of E-cadherin expression.

### Distribution of HIF1α mRNA

**Figure 2:**
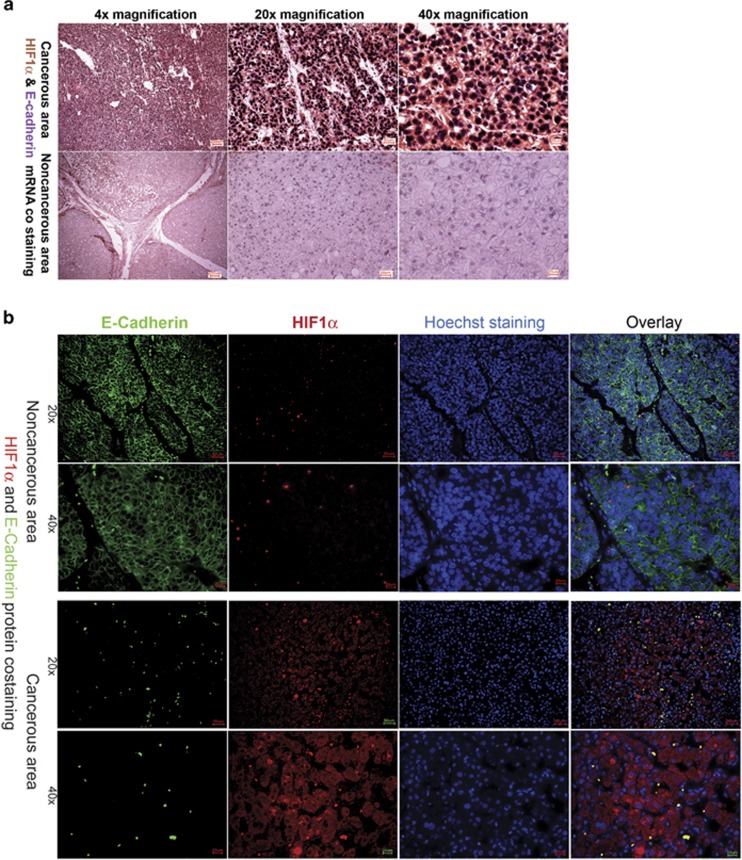
HIF1α and E-cadherin mRNA and protein level distribution in patients tissues. (**a**) Comparative ISH with co-staining for HIF1α (red) and E-cadherin (violet) mRNA expression in CA and NCA sections. Already at the lower magnification an intense nuclear staining for CDH1 mRNA (violet) and a clear cytoplasmic staining for HIF1α mRNA (red) can be seen in CAs, whereas in NCA the signal for HIF1α mRNA (red) is lower but still in the cytoplasm and also the signal for CDH1 mRNA (violet) is lower in nuclei. (**b**) Detection of E-cadherin and HIF1α at the protein level by FIHC in NCA and CAs. Athought E-cadherin protein is highly expressed and visible in cell membranes in NCA, HIF1α protein is highly abundant in the cytoplasm in CAs.

There is strong evidence that one factor influencing E-cadherin levels in cells is the availability of oxygen, as downregulation of E-cadherin and its loss from cell membranes can be observed in hypoxic conditions.^8, 10, 12^ HIF1α is the central regulator of hypoxia-inducible genes. HIF1α is ubiquitously expressed in tissues, and it has been shown that activity of HIF1α is mostly regulated at the protein level.^11^ It also has been shown that basal levels of HIF1α expression in HCCs could predict overall survival of HCC patients.^16^ Moreover, it had been shown that there is an unexpected compensation of cell survival after loss of E-cadherin by overexpression of HIF1α in breast cancer cells.^17^ We therefore wanted to ask whether the basic expression level of HIF1α is similar in CAs and NCAs. In addition, HIF1α is clearly expressed in both CA and NCA sections; the abundance of HIF1α mRNA is much higher in CA sections, in which a very strong cytosolic staining for HIF1α mRNA has been obtained (Figure 2a). This further confirmed that retention of CDH1 mRNA is very specific and not due to a general accumulation of mRNA in the cell nucleus. The strong cytosolic signal for HIF1α mRNA in the cytosol of cells in CAs, in which CDH1 mRNA is only detected in the nucleus, further suggested that HIF1α should be highly expressed in the CA of HCC patients. This is nicely confirmed by comparing protein levels of HIF1α and E-cadherin in tissue sections by FIHC (Figure 2b).

### Quantitative analysis of CDH1 and HIF1α mRNA in CA and NCA samples

**Figure 3:**
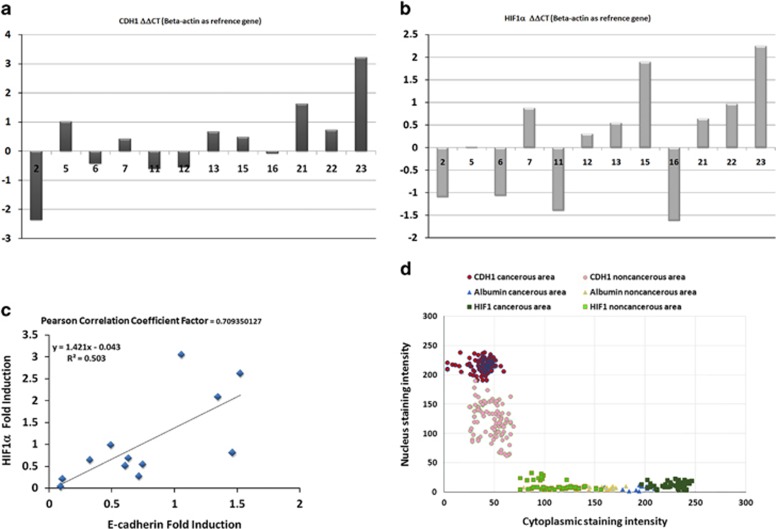
Quantification of CDH1 and HIF1α mRNA and mRNA distribution. (**a** and **b**) Overall levels of CDH1 (**a**) and HIF1α (**b**) mRNA quantified by RT–qPCR from total RNA isolated from CA and NCA tissue sections of 12 patients. The ratio of CDH1 and HIF1α mRNA between CAs and NCAs was calculated as fold induction over the NCA expression levels, showing changes in both directions, increase or decrease in CA vs NCA tissue sections. beta-actin was used as reference for normalization. (**c**) Comparison of the CA vs NCA fold difference of CDH1 and HIF1α mRNA shown in (**a**) and (**b**) for all patient samples. Despite the wide distribution of values (ratios), a pearson correlation factor was calculated for the assumption that E-cadherin and HIF1α differences between CAs and NCAs in HCC patients would correlate.

Because of the striking differences in mRNA signals in cancerous and noncancerous cells, we next asked whether this influences overall mRNA levels of CDH1 and HIF1α in tissue sections representing cancer and cancer-free areas. For this, matching tissue aliquots of either the CAs or the NCAs of all patients were used for RNA extraction. The total RNA obtained was then used to quantify CDH1 and HIF1α mRNA by RT–qPCR using beta-actin for normalization. To our surprise, although CDH1 and HIF1α mRNA levels differ significantly between the matched samples of each patient, no clear distinction between CA and NCA based on the relative mRNA levels is observed (Figure 3a). Interestingly, the ratios of CDH1 mRNA levels of CA and NCA tissue sections include lower, as well as higher, CDH1 mRNA levels in CAs vs NCAs. A quite similar picture is obtained for HIF1α mRNA (Figure 3b). To see whether there is any correlation between CDH1 and HIF1α overall mRNA levels, we compared the ratios obtained of both genes for each patient (Figure 3c). The correlation plot of the CA vs NCA ratios of CDH1 and HIF1α mRNA levels shows quite a wide distribution. Despite of the several samples in the vicinity of the calculated correlation line, the number of outliers present in the correlation plot indicate a low significance, suggesting that there is no correlation between the ratios of these two genes at the mRNA level from CA and NCA tissue sections.

### Semiquantitative analysis of nuclear and cytosolic mRNA levels by ISH image analysis

A reason for these unlcear results analyzing total RNA from tissue samples, despite selection for cancerous and cancer-free sections, is most likely the strong heterogeneity of the liver tissue containing many different cell types, which can mask significant changes occuring in a subpopulation including the carcinoma cells.^18^ As RNA from cytosolic and nuclear fractions of a specific cell population from patient tissue samples could not be prepared, we implemented a semiquantitative image analysis protocol that allows us to determine the relative levels of the nuclear and cytoplasmic abundance of specific mRNAs in ISH images on an individual cell level (Figure 3d). Here the relative signal intensity in the nucleus and cytosol is recorded for each gene at the single-cell level and plotted in a diagram with the *x* axis for cytosolic signals and the *y* axis for nuclear signals. Although CDH1, HIF1α and Albumin mRNA levels are plotted in a single diagram, it should be noted that the values only show the shift for each gene but do not reflect absolute abundance, owing to the lack of an independent reference and variations between the probes for each gene. Nevertheless, the diagram clearly shows that the relative abundance of CDH1 mRNA is signficantly shifted to an increased level in nuclei in CA cells. The signal for HIF1α mRNA is always higher in the cytosol and shifted to an even higher level in CA cells. In contrast, the signals for albumin cluster very close together, and show only a very small increase in the cytosolic signal in CAs. We like to note that it also can be seen that cytoplasmic levels of the CDH1 mRNA are decreased in CA cells. Taken together, the quantitative and semiquantitative analysis of CDH1, HIF1α and Albumin mRNA levels clearly indicate that enrichment of CDH1 mRNA in nuclei of cells in CAs is very specific for this gene and is not seen for HIF1α and Albumin mRNA.

### Cellular distribution of CDH1 mRNA in tumor cell lines and E-cadherin protein levels on the cell surface

**Figure 4:**
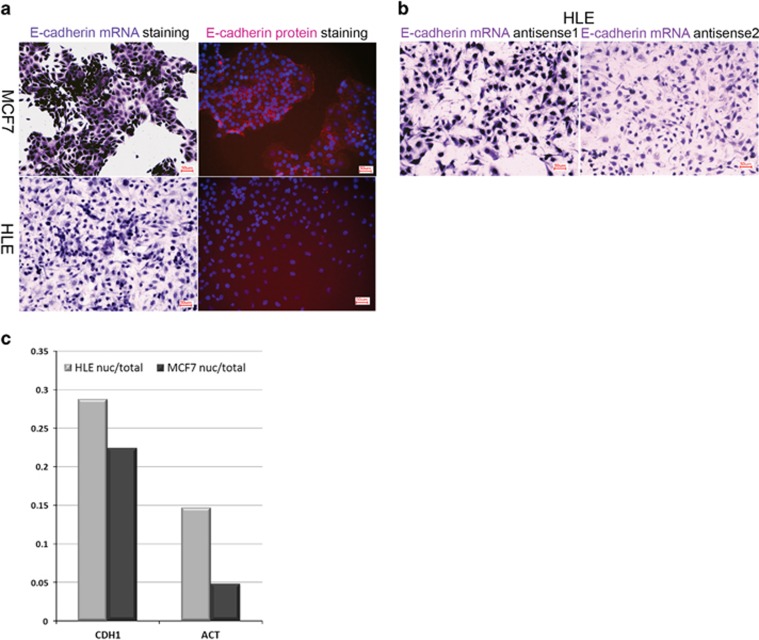
Expression and distribution of E-cadherin in cancer cell lines. (**a**) Staining of CDH1 at mRNA (violet) and protein (red) levels in HLE and MCF7 cell lines revealed that loss of E-cadherin protein signal in HLE cells is directly correlated with a higher signal for CDH1 mRNA in the cell nucleus. In contrast, in MCF7 cells, cytoplasmic CDH1 mRNA levels are high and correlate with high E-cadherin protein levels, resulting in a clear detection of E-cadherin protein localized in cell membranes. (**b**) Comparison of CDH1 staining by using two different E-cadherin antisense RNA probes (Table 1) (violet) in HLE cell line. Probes were designed for the detection of different parts of CDH1 mRNA (coding and non-coding sequences). Independent of the probe used for CDH1 mRNA detection, similar nuclear and cytoplamsic staining is observed. (**c**) Relative distribution of mRNA from nuclear and whole-cell extracts of HLE and MCF7 cell lines analyzed for CDH1 and beta-actin mRNA by RT–qPCR. Detailed values are shown in Table 3.

To see whether the observed nuclear retention of CDH1 mRNA also occurs in other conditions, we looked for cell lines that show different expression levels of CDH1. HLE, a human nondifferentiated hepatoma cell line, in which E-cadherin expression is very low and not detectable in the growth conditions used, and the breast cancer cell line MCF7, which is a well characterized epithelial cancer model and expresses high levels of E-cadherin. We used these two cell lines and performed ISH and fluorescent immunocytochemistry (FICC) for E-cadherin expression using comparable growth conditions. As expected, a very strong signal for CDH1 mRNA was obtained in MCF7 cells with clear staining in the cytoplasm, as well as in the cell nucleus (Figure 4a). HLE cells also showed a signal for CDH1 mRNA, which was, however, completely confined to the cell nucleus. To exclude mostly unspecific binding of the probe, we used two independent CDH1 ISH probes, covering different parts of the gene (Table 2). Both antisense CDH1 probes produced a comparable E-cadherin mRNA staining pattern (Figure 4b), with a clear nuclear signal for E-cadherin mRNA. Further analysis by FICC, using CDH1-specific antibodies (Figure 4a), clearly showed that, at the protein level, E-cadherin could not be detected in HLE cells, whereas a clear signal for E-cadherin was obtained in the cell membrane of MCF7 cells (red fluorescence).

To ensure that CDH1 mRNA is indeed present in HLE cells and to further analyze the subcellular localization of this mRNA, we next isolated total RNA from whole cells, as well as purified nuclei from both HLE and MCF7 cells. Using standard RT–PCR for E-cadherin and beta-actin for reference bands for CDH1, mRNA were obtained with RNA from both cell lines (data not shown). More detailed analysis using reverse transcription and RT–qPCR provided a more detailed picture (Table 3). Although CDH1 mRNA is detected with low threshold cycle (Ct) values in MCF7 cells, confirming a high expression level, in HLE cells the signal is only obtained with higher Ct values in both nucleus and whole-cell extracts, whereas Ct values for beta-actin are more similar in both cell lines (Table 3). We like to note that Ct values for CDH1 and beta-actin mRNA were always higher in RNA extracted from cell nuclei using equal amounts of cDNA in the RT–qPCR. We then asked how these Ct values can be translated into enrichment or depletion of mRNA from the nucleus. We therefore calculated the ratio between nuclear and whole-cell extracts using ΔΔCt to visualize the relative distribution (Figure 4c). As the calculated RE values for the genes analyzed were always significantly lower in nuclear extracts, all ratios are <1, ranging from 0.28 to 0.05, with higher ratios for CDH1 and smaller ratios for beta-actin. For both genes the ratio is always significantly lower in MCF7 cells (Figure 4c). The highest ratio is found for CDH1 in HLE cells, suggesting that retention in the nucleus could be an additional factor ensuring efficient suppression of E-cadherin expression in HLE cells. Although these values do not reflect the steep difference seen in ISH, with only nuclear staining for CDH1 mRNA in HLE cells (Figure 4a); these data confirm that CDH1 is less efficiently transported to the cytoplasm in these cells.

## Discussion

We analyzed E-cadherin expression and cellular distribution at the RNA and protein levels in HCC tissue sections, as well as in two cancer cell lines. It is already well established that E-cadherin expression is lost in solid tumors at the protein level^7, 12, 19^ and that this downregulation of E-cadherin is associated with the development of metastases in cancer patients.^19^ Using ISH and FIHC to detect the cellular distribution of E-cadherin mRNA and protein in tissue sections of HCC patients, we observed two apparently contradicting patterns. While at the protein level E-cadherin was clearly reduced and lost from cell membranes, as described previously, at the mRNA level a strong signal for CDH1 mRNA was obtained in CA of tissue sections, with a striking confinement of the CDH1 mRNA to the nucleus. In contrast, in NCAs, weaker nuclear staining for CDH1 mRNA was observed, which correlated with clear staining for E-cadherin protein on cell membranes (Figure 1a, c and d). Thus, we postulate that nuclear retention and transport to the cytosol is involved in the regulation of E-cadherin expression.

Further analysis in liver and breast cancer cell lines HLE and MCF7, representing a less differentiated and a strong epithelial phenotype, shows that this can also be observed at the cellular level.

In MCF7 cells, a strong signal for E-cadherin protein is detected on the cell surface, and ISH staining for the mRNA resulted in clear cytoplasmic and nuclear signals. In HLE cells, a strong nuclear staining without any visible cytosolic signal for CDH1 mRNA is seen, whereas E-cadherin is not detected at the protein level. Further comparison of nuclear and whole-cell mRNA levels in both cell lines confirmed that CDH1 mRNA distribution is clearly shifted to the nucleus in HLE cells. Interestingly, the more highly expressed gene beta-actin showed a lower nuclear-to-whole cell ratio in both cell lines.

ISH co-staining with E-cadherin and HIF1α antisense mRNA showed that in CAs most of the cells with higher levels of HIF1α mRNA in the cytosol had stronger E-cadherin mRNA staining in the nuclei, suggesting that HIF1α expression is upregulated in tumor tissue. Although no general enrichment of HIF1α could be detected by RT–qPCR in tumor sections; the results obtained by ISH fit well with previous observations that loss of E-cadherin at the protein level could be compensated by HIF1α expression to ensure cell survival^7, 12^ and that the high levels of HIF1α could indicate a poorer prognosis for cancer patients.^16^ This question had not been analyzed closer at this stage, as follow-up data are not available at present.

The major finding of our work is that reduction of the E-cadherin protein could involve retention of the mRNA in the nucleus, which would prevent translation in the cytosol. Although nuclear export is a regulated process, nuclear retention of a specific mRNA has not been described as a regulatory mechanism. A possible alternate explanation could be rapid degradation of cytosolic mRNA, which could lead to a similar picture if the speed of degradation greatly exceeds the speed of nuclear export. Independent of the mechanism, this must be gene-specific, as albumin, HIF1α and beta-actin mRNAs are not effected in the same way.

At the level of gene expression control, our observation suggests that E-cadherin expression is not completely turned off at the gene level,^20^ but the combination of complementary regulatory mechanisms at the mRNA and translation levels is sufficient to ensure complete downregulation when required. Consequently, when cells move to another environment, translation of E-cadherin could be easily resumed and could facilitate anchoring of cells and reestablish epithelial type growth in a new location.

## Materials and methods

### Sample collection

Liver tissue samples were obtained from CAs and NCAs of 25 HCC patients (undergoing surgery) collected since 2011–2014 (Table 1). The study has been approved by the ethics committee of the Medical Faculty Heidelberg of Heidelberg University (Ethikkommission I Heidelberg: Studienzeichen: S-202/2012). All samples were divided in two parts. One part was kept in 4% paraformaldehyde for subsequent embedding in paraffin blocks, and the other part was used immediately for RNA extraction. Diagnoses were established by conventional clinical and histological criteria according to the World Health Organization, and all clinical investigation has been conducted according to the principles expressed in the Declaration of Helsinki.

### Preparation of probes for ISH

Total RNAs were isolated from human MCF7 and HLE cell lines (see below) with the Split RNA Extraction Kit (Lexogen GmbH, Vienna, Austria). First-strand cDNA was synthesized with 3 μg of total RNA using random hexamer primers and Reverse Transcriptase (RevertAid, Fermentas, Sankt Leon-Rot, Germany). Primers for the selected genes were designed as described previously^14^ and antisense RNAs were prepared for E-cadherin (CDH1), covering different parts of coding and noncoding sequences, HIF1α and albumin mRNAs (Table 2). Double-stranded DNA templates were prepared as described.^14^ Digoxigenin and fluorescein-labeled riboprobes were synthesized from purified double-stranded PCR DNA template fragments using the respective labeled nucleotide mix (Roche, Mannheim, Germany) and SP6 or T7 RNA polymerase (Fermentas). Sense and antisense-labeled RNAs were prepared with SP6 and T7 RNA polymerases.

### Preparation of sections for ISH and FIHC

Fresh tumor samples of ~1 cm^3^ were taken from the tumors within 40 min of resection and transferred on ice to the laboratory. Human liver paraffin blocks from different parts of patients' liver (CAs and NCAs) were prepared and cut into 4-μm slices. CAs and NCAs from each patient were mounted on the same poly-l-lysine-coated slides, air-dried overnight (O/N) at 37 °C and stored at 4 °C for 48 h before ISH or FIHC.

### ISH of patient liver sections

ISH was performed for patients' sections with digoxigenin- and fluorescein-labeled antisense probes, as described before.^14^ Two different anti-digoxigenin antibodies coupled either with Cy3 or alkaline phosphatase enzyme were used to detect digoxigenin-labeled RNA probes. Enzymatic detection with anti-DIG-AP was done as described before. For fluorescent detection of probes, 500 μl of Cy3-conjugated anti-digoxigenin antibody (Jackson Immunoresearch Laboratories, Inc., Suffolk, UK) diluted 1:200 in PBS (+10% goat serum and 0.1% tween) was added directly to sections and incubated at 4 °C O/N. Sections were washed with PBS (0.1%) and Hoechst 33342 was used for the staining of nuclei.

### Fluorescence immunohistochemistry (FIHC) of patient liver sections

Paraffin sections were dewaxed with xylol, rehydrated in decreasing ethanol concentrations and washed with deionized water. Antigen unmasking was done with Tris-EDTA buffer (100 mM Tris-Base and 10 mM EDTA pH 9). Sections were washed with Tris-NaCl-Tween (100 mM Trizma base and 150 mM sodium chloride pH 7.5) and blocked with 100–400 μl of blocking solution at room temperature (RT) for 1 h. Anti-Human-E-Cadherin antibody [EP700Y] (Abcam) and Anti-Human-E-Cadherin (67A4) sc-21791 (Santa Cruz Biotechnology) were used as primary antibodies. Around 200 μl of diluted antibodies (1:500 in blocking solution) were added to sections and incubated at 4 °C O/N. The day after, antibodies were removed and sections were washed with wash buffer (Tris-NaCl-Tween buffer). Alexa Fluor 594-conjugated AffiniPure Goat Anti-Rabbit IgG and Alexa Fluor 488-conjugated AffiniPure Goat Anti-Mouse IgG were used as secondary antibodies. A volume of 200 μl of diluted secondary antibody (1:500 in blocking solution) was added and slices were incubated at RT for 2 h. After washing with PBS two times for 10 min and nuclear staining with Hoechst 33342, sections were mounted with mowiol.

### Cell lines and FICC and ISH in cell lines

HLE (obtained from JCRB, no: JCRB0404) is a nondifferentiated human hepatoma cell line and is grown in monolayer adherent culture. MCF7 (obtained from CLS, No: 300273 Vital: 330273) is a epithelial-like, breast adenocarcinoma, human cell line and maintained in monolayer adherent culture.

Altogether, 10^4^ cells (MCF7 or HLE) were suspended in 200 μl of medium (DMEM with penicillin/streptomycin and 10% (v/v) FCS) and seeded in μ-slides eight-well plates (Ibidi GmbH, München, Germany). Plates were incubated in a standard tissue culture incubator at 37 °C, 5% CO_2_ and 95% humidity for 48 h. Cells were washed with cold PBS and fixed with 4% paraformaldehyde. Before ISH or FICC was started, cells were washed with PBS one more time. For ISH, cells were incubated with 20 μg/ml proteinase K at 37 °C for 4 min and processed as explained earlier^14^ with probes listed in Table 1. For FICC, cells were permeabilized with 0.2% Triton-PBS (RT, 10 min), washed with PBS and blocked with blocking buffer (2% goat serum in PBS, RT 1 h). Cells were washed with PBS, and 200 μl of diluted primary antibody (1:500 in 2% goat serum in PBS) was added to all wells. Plates were incubated at 4 °C O/N. Next, cells were washed with PBS and diluted secondary antibody was added to all wells. Plates were incubated (RT, 1 h) and washed with PBS. Nuclei were stained with Hoechst 33342 and pictures were taken using a microscope (BZ-II Analyzer, Keyence, Neu-Isenburg, Germany).

### RNA extraction from human tissue, cell lines and cell nuclei

The Split RNA Extraction Kit (Lexogen GmbH, Vienna, Austria) was used for RNA extraction from patients liver tissues (CAs and NCAs separately) and cell lines.

### For preparation of whole cell and nuclear RNA

A total of 10^6^ HLE (JCRB cell bank: JCRB0404) or MCF7 (ATCC: HTB-22) cells were seeded in medium-sized flasks for 48 h until 80–90% confluence was reached (10^7^ cells). Cells were washed with cold PBS and collected from flasks. They were suspended in PBS and split into two parts, and mRNA was extracted from the first part of collected cells with the RNA isolation columns. The remaining cells were washed with cold PBS and centrifuged at 1500 r.p.m. for 1 min. The cell pellets were suspended in 400 μl of ice-cold buffer A (Hepes 10 mM, KCl 10 mM, EDTA 0.1 mM and DTT 1 mM, pH 7.9, in 100 ml water) and incubated for 10 min on ice; 25 μl of NP-40 Alternative10% was added to the mixtures and they were vortexed for 10 sec. The suspensions were centrifuged at 9000 r.p.m. for 1 min. Supernatants were removed, nuclear pellets were mixed for 10 sec with 500 μl of buffer A and 20 μl of NP-40 Alternative10% and centrifuged again at 9000 r.p.m. for 1 min; the supernatants were discarded. Nuclear pellets were then used for RNA extraction.

### Real-time RT–qPCR

A measure of 1118 ng of total RNAs either isolated from human tissues or whole-cell lysate and their nuclei were used for cDNA synthesis and quantitative real-time PCR on the Real-Time PCR-Thermocycler qTOWER (Analytik Jena AG, Jena, Germany) machine. 2 μl of cDNA (1:20 dilution of transcribed cDNA), 5 μl of LightCycler 480 SYBR Green I master mix (Roche GmbH, Mannheim, Germany), 1 μl of respective diluted PCR primers (1:20) (Table 2) and 2 μl of water were used. RT–qPCR was performed using the following protocol: 1 cycle of preincubation for 5 min at 95 °C, followed by 40 amplification cycles for 10 s at 95 °C, 10 s at 60 °C and 20 s at 72 °C. For all samples, a melting curve analysis was performed in order to monitor the generation of the expected unique PCR products. For statistical analysis, relative gene expression (RE) levels were calculated with the function (RE=2^−^ΔΔCt), where ΔΔCt is the normalized difference in threshold cycle number of the CA samples and the NCA samples. Each Ct value was calculated from triplicate replicates of any given condition. All samples were normalized to the corresponding expression level of beta-actin. cDNAs synthetized from mRNAs extracted either from nucleus or whole-cell lysate of HLE and MCF7 cells were used for E-cadherin and beta-actin RT–qPCR (Table 3); relative expression normalized to highest expression for each gene was calculated (Figure 4c).

### Semiquantitative evaluation of nuclear and cytosolic mRNA levels by ISH and image analysis

ImageJ (NIH resources - http://imagej.nih.gov/ij/) was used to record the signal intensity of mRNA staining from ISH images in nuclei and cytoplasm of 10 random selected cells per patient from CAs and NCAs. All images were processed in the same manner. For CDH1, images from 10 patients were used; images from five patients were used for HIF1α and images from three patients were used for the analysis of Albumin mRNA distribution (Figure 3d).

## Figures and Tables

**Table 1:** Patient information

*Gender*	*Age*	*Diagnose (pathology)*	*Tumor size (pathology)*	*Liver disease*	*Therapy*	*Tumor stage TNM*
M	60	HCC	2.5 cm	HCV infection	2 × TACE	—
M	25	HCC	1 × 3 × 2 cm	HBV infection	—	pT2, PN0, V1, R0
F	77	HCC	5 × 5.7 × 6.3 cm	Cryptogenic liver cirrhosis	—	pT1, pN0, G2
M	70	HCC	—	Alcoholic liver cirrhosis	—	—
M	79	HCC	1.0 × 1.0 × 0.8 cm	Alcoholic liver cirrhosis	3 × TACE	pT1, pN0, pM1, G2
M	57	HCC	2.2 × 1.5 × 3 cm	HBV infection	1 × TACE	pT3, pN0, M0
M	76	HCC	1.4 × 1.9 cm	HBV infection	—	pT1, pN0, G1
M	82	HCC	—	—	—	—
M	38	HCC	—	—	Nexavar	—
M	68	HCC	5.3 × 4 × 4 and 1.2 × 1.2 cm (2 tumors)	Alcoholic liver cirrhosis	—	pT2, G3
M	53	HCC	3.5	HBV, HCV (alcoholic liver cirrhosis)	—	pT2, pN0, M0, G2
M	76	HCC	3.6	HBV (alcoholic liver cirrhosis)	—	pT2, N0, M0, G3
F	33	HCC–CCC	2.3	Autoimmune hepatitis	Nexavar	pT1, N0
M	57	HCC	—	HCV infection	—	—
M	63	HCC	14	HCV infection	—	pT3a, pN0, G2
M	63	HCC	2.2	Alcoholic liver cirrhosis	—	PT1 G2
F	75	HCC	3	HCV+HBV infections	—	pT2, PN0, G2
M	66	HCC	2.5	Alcoholic liver cirrhosis	—	Pt2, PN0, G2
M	60	HCC	2.5	HBV infection	—	pT1, G2, R0
M	69	HCC	—	Alcoholic liver cirrhosis	4 × TACE	rpT1, N0, G2
M	68	HCC	3.2	—	—	pT1, pN0, G2
M	77	HCC	0.5	—	—	—
F	71	HCC	2.7	Steatohepatitis	—	PT3b, G2
F	77	HCC	3.7	Steatohepatitis	—	PT1, PN0, G2
M	77	HCC	5	Hemochromatose	—	PT2, PN0, G2

Abbreviations: CCC, cholangiocellular carcinoma; F, female; HBV, hepatitis b virus; HCV, hepatitis c virus; HCC, hepatocellular carcinoma cell; M, male.

**Table 2:** Primer sequences and the length of each antisense or qRT–PCR products

*Gene*	*Reference sequence*	*Method*	*Length*	*Forward*	*Reverse*
E-cadherin	NM_004360.3	ISH	910^a^	5′-CCCGCCTTATGATTCTCTGCTCGTG-3′	5′-CTGTAATCCCAATACTCTGGGAGGC-3′
			884^b^	5′-GTGACAGAGCCTCTGGATAGAGAAC-3′	5′-GCAGTGTAGGATGTGATTTCCTGGC-3′
		qRT–PCR	160	5′-CCCGCCTTATGATTCTCTGCTCGTG-3′	5′-TCCGTACATGTCAGCCAGCTTCTTG-3′
HIF1α	NM_001243084.1	ISH	837	5′-CATGGAAGGTATTGCACTGCACAGG-3′	5′-CAGCACTACTTCGAAGTGGCTTTGG-3′
		qRT–PCR	189	5′-CATGGAAGGTATTGCACTGCACAGG-3′	5′-TCATATCCAGGCTGTGTCGACTGAG-3′
ACTB	NM_001101	qRT–PCR	201	5′-CTGACTACCTCATGAAGATCCTC-3′	5′-CATTGCCAATGGTGATGACCTG-3′
Albumin	NM_000477	ISH	1403	5′-GGTGAGACCAGAGGTTGATGTGATG-3′	5′-CACACATAACTGGTTCAGGACCACG-3′

Abbreviations: HIF1α, hypoxia-induced factor-1α ISH, in s*itu* hybridization; qRT–PCR, quantitative reverse transcription–PCR.

a E-cadherin antisense RNA1.

b E-cadherin antisense RNA2.

**Table 3:** E-cadherin and beta-actin Ct values for different RNA sources

*Extracted RNA*	*E-cadherin cycle threshold*	*Beta-actin cycle threshold*
HLE nucleus	26.8	19.3
HLE whole-cell lysate	25	16.53
MCF7 nucleus	19.6	18.33
MCF7 whole-cell lysate	17.44	13.95

Abbreviation: Ct, threshold cycle.

## Abbreviations

HCC: hepatocellular carcinoma cell
HIF1α: hypoxia-induced factor-1α
FIHC: fluorescent immunohistochemistry
ISH: *in situ* hybridization
CA: cancerous area
NCA: noncancerous area
