# Supplementary figures and images for: Nuclear accumulation of CDH1 mRNA in hepatocellular carcinoma cells
Source: Oncogenesis. 2015 Jun 1;4(6):e152–. doi: 10.1038/oncsis.2015.11 (PMC4753520; doi:10.1038/oncsis.2015.11)

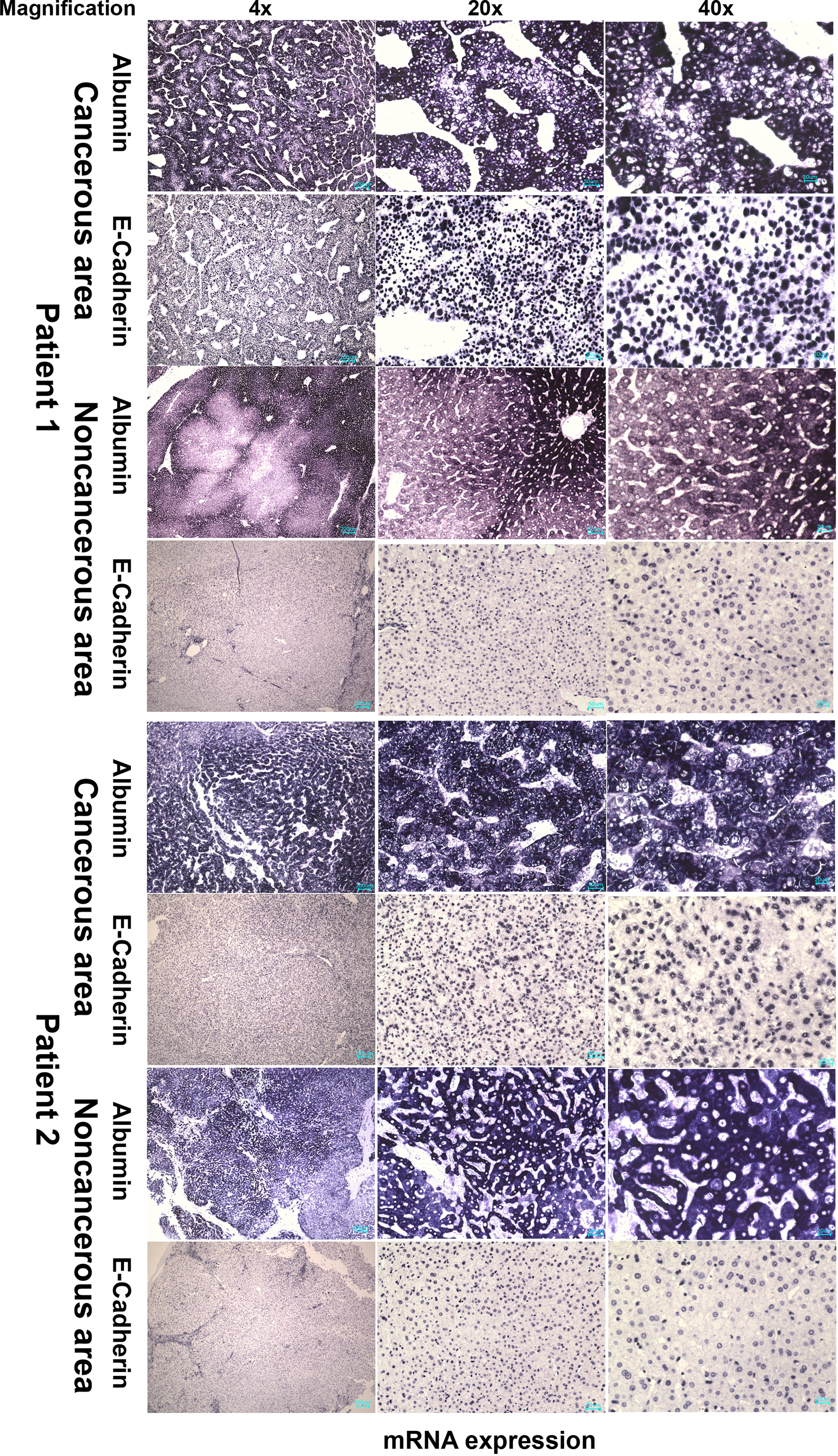

Supplement: Supplementary Figure S1 [file oncsis201511x1.tif]
