# Supplementary material for: Nuclear accumulation of CDH1 mRNA in hepatocellular carcinoma cells
Source: Oncogenesis. 2015 Jun 1;4(6):e152–. doi: 10.1038/oncsis.2015.11 (PMC4753520; doi:10.1038/oncsis.2015.11)
Supplement: Supplementary Figure S1 Legend [file oncsis201511x2.doc]

**Supplementary Figure1:**

**In support of Figure 1 higher magnification images with staining for CDH1 and Abumin mRNA (violet) as indicated are shown for cancerous (CA) and noncancerous areas (NCA) from two HCC patients:** In situ hybridization (ISH) shows CDH1 and Albumin mRNA levels and distribution. In both patients the staining for Albumin mRNA is cytosolic in both, CA or NCA, hepatocytes, whereas CDH1 showed a strong retention in hepatocyte nuclei in CA and less nuclear retention in NCA.

**Abbreviations:**

HCC: Hepatocellular Carcinoma Cell, HIF1α: Hypoxia-Induced Factor1α, FIHC: Fluorescent Immunohistochemistry, ISH: *In Situ* Hybridization, CA: Cancerous Area: NCA: Non-Cancerous Area
